# Ethics of Buying DNA

**DOI:** 10.1007/s11673-022-10192-w

**Published:** 2022-07-19

**Authors:** Julian J. Koplin, Jack Skeggs, Christopher Gyngell

**Affiliations:** 1 Melbourne Law School, University of Melbourne, 185 Pelham St, Carlton, VIC 3053 Australia; 2 Biomedical Ethics Research Group, Murdoch Children’s Research Institute, Melbourne, Australia; 3 Monash University, Melbourne, VIC Australia; 4 Department of Paediatrics, University of Melbourne, Melbourne, VIC Australia

**Keywords:** Genomics, DNA databases, Direct-to-consumer genetic testing, Commodification, Exploitation

## Abstract

DNA databases have significant commercial value. Direct-to-consumer genetic testing companies have built databanks using samples and information voluntarily provided by customers. As the price of genetic analysis falls, there is growing interest in building such databases by paying individuals for their DNA and personal data. This paper maps the ethical issues associated with private companies paying for DNA. We outline the benefits of building better genomic databases and describe possible concerns about crowding out, undue inducement, exploitation, and commodification. While certain objections deserve more empirical and philosophical investigation, we argue that none currently provide decisive reasons against using financial incentives to secure DNA samples.

Since 2005, direct-to-consumer (DTC) genetic testing companies have offered consumers the opportunity to have their genomes analysed (Allyse et al. 2018). Unlike testing in a clinical setting, DTC genetic testing involves individuals purchasing genetic tests and receiving results without the involvement of a healthcare professional (Skirton et al. 2012). For a price, DTC companies will extract genetic data from an individual’s saliva sample and generate reports about their genetic heath and/or ancestry.

This price is rapidly falling.

DTC companies originally charged consumers upwards of US $1000 for their services. Today, consumers can have their data analysed for less than US $100. While the reduced cost of genomic sequencing helps explain this fall, the increasing value of genetic databases may contribute to these prices falling further. Researchers have used the database of DTC company “23andMe” to investigate novel genetic variants associated with Parkinson’s disease, demonstrating the potential scientific and financial value of DTC databases (Mullard 2015). In 2018, the pharmaceutical company GlaxoSmithKline paid 23andMe US$300 million for the rights to use its database to search for genetic associations with disease (Regalado 2016). The largest DTC Company, Ancestory.com, has reached a similar agreement with google subsidiary California Life Company, in this case to enable research on the genetics of ageing (Leavenworth 2018).

The maintenance of genetic databases is already central to some DTC-GT companies’ business model (Roberts, Pereira, and McGuire 2017; Stoeklé et al. 2016). As the financial value of genetic databases increases, DTC companies are able to charge customers less in exchange for access to their genetic information. We are likely to soon reach a point where companies will be motivated to pay consumers for their DNA (Roberts et al. 2017). Indeed, some companies have already begun to do so. In 2018, DTC company Luna DNA was the first to actively pay individuals for their DNA and personal data (Tracer and Brodwin 2018). Similarly, Nebula Genomics has plans to establish an online marketplace where users can exchange access to their genetic information to earn “Nebula tokens,“ a form of cryptocurrency (Ahmed and Shabani 2019). Other companies—such as EncrypGen—aim to broker exchanges between individual data sellers and buyers (DeFrancesco and Klevecz 2019). Still others—such as Genos and Invitae—are considering selling customers’ genetic information and sharing the profits with those who contributed their information (Roberts et al. 2017).

Some companies are considering offering incentives that, while not strictly payment, nonetheless have financial value. Nebula Genomics plans to offer free personal genome testing to consumers who agree for their heath data to be shared with or sold to third parties (Regalado 2018), while 23andMe has previously recruited patients with specific disorders by giving its test away for free (Regalado 2016). While such incentives do not constitute buying DNA per se (and therefore fall largely outside the scope of this paper)*,* it is worth noting that offering incentives with clear monetary value would likely raise many of the same ethical issues as offering direct cash payments.

To date, ethical analyses of DNA markets have largely focused on sale of services to consumers (see, e.g., Allyse et al. 2018; Caulfield and McGuire 2012; Mena and Terry 2017; Vayena 2015). There has been little ethical analysis of paying individuals for their DNA and personal data.^1^ We aim to remedy this gap.

We begin from the position that there are legitimate moral questions about what kinds of goods ought to be traded via the market. We assume that it is appropriate for some things to be traded via the market and for some things to be excluded from market exchange.

It is worth acknowledging two opposing accounts of the moral limits of markets: market abolitionism and market universalism (Walsh 1998). The first—market abolitionism—holds that markets should be abolished altogether. Market abolitionism would rule out the sale of “contested commodities” like kidneys and genetic data but also regular market commodities like eggplants or eiderdown pillows. The second view—market universalism—accepts the commodification of any and all goods that can be traded via markets. Market universalism would endorse DNA markets for precisely the same reason it endorses market exchange in pot plants, human organs, or any other commodifiable good.

Neither market abolitionism nor market universalism can help answer this paper’s motivating question: whether there is anything especially problematic about DNA markets, relative to other kinds of markets we generally accept. We are interested in whether those who accept some (but not all) markets ought to also accept markets in DNA. This task will require us to weigh the moral costs and benefits of paying individuals for DNA and personal data.

## Benefits: The Need for Better Genomic Databases

As seen above, databases built by through DTC companies are already contributing to medical research. They can potentially solve much wider problems. Most complex disease like cancer and heart disease are affected by many genes, each of which has a small effect (see, e.g., Khera et al. 2018). Genetic variants that have only a small effect on a trait, or that are only present in a small section of the population, will only be detectable in studies with very large sample sizes. The fact that DTC companies already have samples from millions of users means they could help us understand the impact of genetic variation on chronic disease and its interaction with environmental risk factors.

However, in their current state DTC databases have a major limitation—a lack of genetic diversity. Over 75% of 23andMe’s customers are of European ancestry, a group that accounts for only a small share of the world’s population (Dickey 2018). This limitation is also shared by major clinical databases. One of the most widely used resources for genetics research, UK Biobank, contains samples from over half a million people, 95% of whom are of European ancestry (Bycroft et al. 2018). It is now widely believed that a key to improving genetics research is to improve diversity in databases (Korlach 2019). Not only will this improve the reliability of research, it will remove a major obstacle to achieving a fairer distribution of the benefits of genomic research among ethnic groups.

Paying for samples is one way that DTC companies could attract more diverse customers. Companies could adopt differential pricing structures, allowing them to attract genomes needed most. This could include, for example, paying a higher premium for those who are currently underrepresented in genomics databases, such as those from African, Asian, Pacific Islander, or Indigenous ancestries. It could also include paying higher amounts for genomes of people with family histories of disease whose genetic contributions are still unknown.

At this juncture, it is important to note that genomic research with Indigenous peoples raises important ethical issues. There is a burgeoning literature on how best to engage with Indigenous communities across protocol development, participant recruitment, and data management (Claw et al. 2018; Garrison et al. 2019; Kowal 2012; Tsosie, Yracheta, and Dickenson 2019). It is possible that such engagement might rule out payment in some communities—for example, those where it is believed that DNA is not property and should not be traded on the market. What is doing the work here, however, is not a general objection to payment, but rather a commitment to taking the values of Indigenous communities seriously. Having noted that payment might not be appropriate in such contexts, we will leave this issue to one side. In what follows, we are interested in whether there are any legitimate objections to paying for genetic data more generally (not just when interacting with groups that are opposed to payment).

A secondary, ancillary benefit of shifting DTC companies to a pay-for-sample model is that doing so may improve transparency of the current system. In the current model—where consumers pay to receive ancestry and/or medical information—it is not necessarily clear to consumers that their genetic information will be sold to third parties. This raises concerns both about whether DTC-GT consumers give appropriately informed consent and whether the sale of personal information will provoke a public backlash against genomic data-banking by private companies (Stoeklé et al. 2016).^2^ Indeed, a 2016 survey of DTC-GT consumers suggests that many consumers falsely believe that DTC-GT companies will share results only with them, when in fact the company in question uses the information for secondary purposes including research (Christofides and O’Doherty 2016). Shifting from a model where the donor pays to receive ancestry and health-related information to one where they are paid for their donation may help customers understand that genetic and personal data has financial value to DTC-GT companies (otherwise, why would the company purchase it?). Payments thus provide one potential avenue for helping secure customers’ informed consent to the sale of their genetic and personal data.

## Concerns

DNA markets where individuals are paid for their donations will raise many of the same concerns as DNA markets in which genetic tests and/or genealogy services are sold direct to consumers (and consumers’ genetic data is then sold to third parties). Direct-to-consumer companies have been criticized for failing to provide customers with adequate information on how data would be shared, the risk of re-identification of data, and the kinds of research and commercial purposes for which this data is sometimes used (Christofides and O’Doherty 2016; Laestadius, Rich, and Auer 2017; Niemiec and Howard 2016). The donation of DNA samples does carry some risks, including an inherent risk of re-identification and the possibility of regret if the sample is used in research the donor disapproves of (Laestadius et al. 2017). The re-use of customers’ samples in whole genome sequencing (which might turn up further health-related information) raises further questions about whether/how incidental findings should be returned to customers who provided these samples (Adam and Friedman 2016). Many of these issues will also be relevant to business models where individuals are paid for their data.

Since there is an established body of literature on these issues, we will not address them further here. We will instead focus on concerns specifically raised by allowing companies to pay for donations, including the risk that markets will “crowd out” altruistic donation, concerns about undue inducement and exploitation, and the potential for wrongful commodification.

## Motivational Crowding

In other human tissue markets, particularly for blood, many commentators worry that economic incentives might “crowd out” altruistic motivations to donate, potentially reducing overall supply (see, e.g., Bowles 2008; Chell et al. 2018; Sandel 2012). Participants in DNA markets are currently driven by a range of motivations, including a desire to know about genetic risks and ancestry data and a desire to contribute to medical science. The risk here is that if DNA is seen or treated as a commodity, fewer DTC-GT customers might be willing to donate it altruistically.

Another way these concerns could manifest is if payments have a detrimental effect on participation in national genomics programmes. Over US $4 billion has been spent by governments worldwide to establish national genomic medicine initiatives. Over the next five years, genomic data from over sixty million patients are expected to be generated through clinical practice (Stark et al. 2019). These individuals will not be paid for their samples, but rather asked to donate their data. Many individuals choose to participate in these programmes for altruistic reasons, like a desire to further medical research. In a world where one’s genetic information has a price on it, people might be less willing to donate their data to national programmes.

The prospect of motivational crowding raises two distinct concerns. The first is simply that paying for DNA might reduce altruistic donations and thereby prove counter-productive. The second is more complicated. It is based on the idea that we have broader reasons to prefer altruistic donation to the use of financial incentives. Writing in relation to blood procurement, Richard Titmuss (1970) and Peter Singer (1973) have argued that altruistic donation—unlike paid donation—can foster altruism and a sense of solidarity at the societal level. Under an altruistic system, donors give a “priceless” gift—one which has no monetary equivalent—to strangers they will probably never meet. Titmuss and Singer see this practice of gift-giving as a valuable means of sustaining a sense of solidarity and social integration within a political community. Since solidarity and social integration seem to be good things—inter alia, because they can help promote individual and collective wellbeing—they recommend that blood be sourced exclusively from unpaid donors.

Does motivational crowding present a serious risk for DNA markets? We think this question deserves careful study. It is not farfetched to think that payments might dissuade some DTC-GT customers from donating their data altruistically. Notably, the news that 23andMe had sold access to their genetic database to GlaxoSmithKline has already prompted some degree of backlash, with some of the relevant media coverage instructing consumers on how to delete their data and rescind consent for future research (see, e.g., Brodwin 2018). Given this backdrop, one might reasonably worry that further commodifying DNA (by introducing financial incentives to donate) would make some people less willing to donate altruistically.

However, the risk posed by motivational crowding should not be overstated. It might be possible to attract a greater number of donations overall by paying for DNA *even if* these payments crowd out some unpaid donations. Payments might provide an especially useful mechanism for DTC-GT companies to attract donations from groups that are of specific interest to researchers, such as under-represented ancestry groups, but do not tend to make use of DTC-GT services—which might be difficult to achieve without leveraging financial incentives. This prospect should be investigated further.

If payments for genetic data make people less likely to participate in national genomic research programmes, then this could generate reasons against this practice. For one, private companies may not share data widely with research groups. This could reduce the value of such databases as a research tool. Furthermore, the priorities of private companies are less likely to track what is ethically important than national research programmes. Whereas national programmes might have obligations to respond to identified health needs of underprivileged groups, private companies will have no such obligations. We can thus expect private companies to focus their research efforts on applications which maximize profit rather than serve the greatest medical need.

It might be bad for the locus of genomics research to shift from national programmes to private companies. Still, this concern would not, by itself, justify a ban on companies offering payment for genetic data. It could be that companies paying for genetic data has little effect on altruistic motivations to participate in national programmes, especially if, as expected, the amount paid for genetic data is relatively minimal; one estimate places the potential price at roughly US $21 (Tracer and Brodwin 2018). Furthermore, if the act of paying for genetic data increases participation in genomics research, then national programmes could theoretically also offer individuals small payment, either direct or in-kind, as a way of boosting research participation.

Admittedly, this possibility raises another concern. Paying providers of genetic data would raise the cost of developing and maintaining genetic databases. If this increased cost is passed on to researchers, it might become harder for researchers without significant money to conduct important forms of research. While we think this possibility deserves further study, we think it is not a foregone conclusion that payments would hamper research. First, it is unclear how significantly a (potentially quite small) payment would affect overall costs to national programmes. Second, in the case of national programmes, the costs of paying providers need not be passed on to researchers. There is already precedent for such an approach; UK Biobank, for example, charges access fees that track only the costs of servicing an access application, not for developing and managing the data resource as a whole (UK Biobank 2021). Access fees could be further reduced for certain categories of research. Third, even if payments to DNA providers do increase costs to researchers, they may also increase the diversity and quality of genetic databases (see our above discussion of the benefits of payment.) Any increase in cost might be offset by an improvement to the product and thus increase the overall-cost effectiveness of national genomics programmes.

We have so far addressed the pragmatic concern about motivational crowding: that offering payment might prove to be counter-productive. There is, however, a second component to Titmuss and Singer’s argument: that altruistic donation is preferable to paid provision because the former kind of system allows donors to express altruism and solidarity and in so doing might help promote the values of altruism and solidarity on a societal level (Archard 2002; Koplin 2015; Sandel 2012). In this respect, altruistic donation of one’s genetic data might indeed be morally preferable to a market system.

While we acknowledge that concerns about altruism and solidarity provide legitimate reasons to favour an altruistic system, we also want to note some limits to this argument’s force. Writing in a different context, Jeremy Shearmur (2015, 124) has argued that Titmuss saw “a happy coincidence, in forms of blood provision, between telling economic and medical arguments for voluntary [unpaid] blood provision, and issues of social solidarity”; in effect, issues of social solidarity effectively reinforced arguments that unpaid donation was safer and cheaper than a paid system. In the case that concern for solidarity conflicts with these other values—if, for example, payment were necessary to secure an adequate supply of blood—the case for altruistic donation seems much weaker: “blood products… are, in the face of current needs, matters of some real moral urgency; while social solidarity seems not only less pressing, but something which we can address in many other ways“ (Shearmur 2015, 214). In the case of genomic research, we are happy to concede that considerations of altruism and solidarity may provide legitimate grounds to prefer unpaid donation over paid provision, all else being equal. If, however, payment would provide a valuable mechanism to meet currently unmet needs (for example, for genomic data from under-researched groups), then a Titmuss-style case for unpaid donation would, we think, appear rather strained.^3^

## Corruption

One might worry that financial incentives can affect not only people’s willingness to trade that particular good, but also the character or quality of that which is being produced. The concern here—which is sometimes described as a “corruption concern” (Sandel 2012)—is that subjecting something to the norms of the market can result in goods that are of lower quality, or carry greater risk, than if they had been kept outside the market’s domain.

One influential objection to commercial markets in blood takes this form. In 1970, when the United Kingdom was considering introducing paid blood donation, Richard Titmuss (1970) compared the quality of the blood procured under the United Kingdom’s altruistic system and the commercial system in the United States. The blood supplied from unpaid donors in the United Kingdom appeared to be much less likely to carry blood-borne illnesses than the blood supplied from paid donors. One of the explanations Titmuss posited for this discrepancy was that paid donors—especially those badly in need of money—are less likely to give a complete and truthful medical history when doing so might disqualify them as donors. More recently, behavioural economists have explored how financial incentives affect performance on a range of tasks, including IQ tests (where some participants are paid for each correct answer.) Interestingly, offering small financial incentives actually led to worse performance, presumably because these extrinsic incentives “crowded out” intrinsic motivations to perform well (Gneezy and Rustichini 2000). To put this finding in terms of the corruption objection: in the context of IQ test performance, offering (modest) incentives can compromise the quality of the good being produced (in this case, an accurate indication of participants’ abilities.)

In a similar vein, one might worry that paying for genetic data might lead to lower-quality personal data than if donations were motivated purely by the desire to advance science. The amount of care one takes when completing medical records, recording personal history, or providing other relevant information might depend on whether one is motivated by advancing science or by receiving a payment after finishing filling in the data. This is an important concern, and it ought to be monitored carefully. However, it is worth noting that corruption concerns might be amenable to market design; the quality of the information that sellers provide might be susceptible to manipulation with the right incentives. For instance, many companies are now looking at giving rewards not only for the DNA samples, but also according to the type and volume of personal data individuals provide. While corruption concerns do not currently provide a decisive objection to DNA markets, we think it is worth conducting further research on how different forms of incentives affect the quality of the data that sellers on a DNA market provide.

## Undue Inducement

It might be worried that financial incentives would pose an “undue inducement” to sell one’s genetic and personal data. Similar concerns have also been raised with regards to paid participation in biomedical research (Wertheimer and Miller 2008) and markets in human tissues such as solid organs (Cohen 2014) or human eggs (Hyun 2006).

Concerns about undue inducement can take one of two forms. First, it might be thought that incentives can undermine the quality of sellers’ consent by distorting their assessment of the risks and benefits of participating—for example, if they focus myopically on the benefits and disregard the risks (Wertheimer and Miller 2008). Second, it might be thought that there is there is something problematic about using incentives to motivate people to do something to which they are highly averse (such as participating in research they perceive as humiliating or excessively dangerous). Such incentives are sometimes thought to be “undue” in the sense that they fail to respect the targets’ values, beliefs, and preferences (Grant and Sugarman 2004; London 2005). In both cases, concerns about undue inducement are linked to the nature and degree of the risks; they presuppose that the research in question carries more than trivial risks that participants can reasonably disregard.

Do concerns about undue inducement provide reason against paying for genetic information? Although we do not rule out this possibility altogether, we see three demanding obstacles that such an argument would have to clear. First, it would have to be shown that “undue inducements“ are, in fact, a moral problem. This is a bigger topic than we can address in this paper. However, it is worth noting that many philosophers are critical of the idea that financial inducements can be morally problematic. Julian Savulescu (2001), for example, has argued that offering financial inducements for risky research is no different, morally, to offering a high salary for dangerous forms of work, while Ezekiel J. Emanuel (2005) has argued that any ethical research study (in which, inter alia, any risks are counterbalanced by potential benefits) cannot be rendered unethical by the mere addition of a financial incentive. The first challenge, then, is to show that concerns about “undue inducements” are in fact sound.

The second hurdle is to show that, in the case of DNA markets, the risks to sellers are substantial enough for concerns about undue inducement to apply. The key risks here have been cashed out in terms of genetic privacy; complete de-identification of genomic data may not always be possible (Ahmed and Shabani 2019). However, the scope, value, and importance of genetic privacy is still being debated, and on at least some views genetic privacy has no intrinsic and little instrumental value (Goodman 2016). For concerns about undue inducement to hold sway, it must be the case that the risks to sellers are more than minimal.

The third hurdle is to show that the kinds of incentives on offer will, in fact, be sufficiently attractive to pose a meaningful inducement. In research ethics, it is commonly argued that concerns about undue inducement can be met by keeping the size of the incentive small. The idea here is that since modest incentives will not be deeply tempting (except, perhaps, for those in dire straits), they are unlikely to prompt irrational decision-making, distort people’s risk assessment, or cause people to overcome deep-rooted aversion (e.g., London 2019; Macklin 1981). The precise threshold is, however, often left vague. The Council for International Organizations of Medical Sciences (CIOMS), for example, specify in their guidelines only that payments should not be “so large as to induce potential participants to consent to participate in the research against their better judgement,” given the particular social and economic context of the population in question (CIOMS 2016, 54). On this kind of view, concerns about undue inducement only provide reason to reject payments for genetic data if they are large enough to distort participants’ decision-making. The kinds of payments currently on offer—such as the US $21 proposed by LunaDNA (Tracer and Brodwin 2018)—arguably fall below the relevant threshold, and if necessary could potentially be reduced further in order to avoid undue inducement.

## Exploitation

Exploitation concerns are generally linked to the idea that exploitees receives less than they deserve*.* The idea of *unfairness* is central to exploitation*;* to exploit someone is to take unfair advantage of them (Mayer 2007; Zwolinski and Wertheimer 2017).

It is sometimes argued that DTC-GT companies exploit their customers by *failing* to pay them for their data, given the financial value of this information (Fox 2018). On this view, offering payments would be less exploitative than the status quo. However, we do not think there is anything *inherently* exploitative about donations where only one party benefits. We are not necessarily exploited if we give a gift to a loved one without expecting the gift to be reciprocated, or if we donate money to charities that provide services we will not personally benefit from. By the same token, DNA donors are not necessarily exploited if they freely donate their data. They might, however, be exploited if they are offered payment of a size that is in some sense unfair.

How, then, should we understand exploitation? Probably the most influential account of exploitation has been developed by Alan Wertheimer (1996). For Wertheimer, exploitation consists in paying somebody less than they deserve, relative to some normative baseline for a fair transaction. One possible baseline—the one favoured by Wertheimer—is the price that would be agreed upon by two well-informed actors in a competitive market.^4^ On this account of exploitation, DTC companies exploit sellers if they offer payments that fall below this competitive market baseline. Exploitation (so understood) is not *essential* to the market; it can be remedied by ensuring that sellers are paid a fair price.

Wertheimer’s account of exploitation might be too permissive. When the background conditions to a transaction are unfavourable or unjust, the competitive market price might be very low. Anchoring our “fairness baseline” to the competitive market can have counter-intuitive implications. For example, it would suggest that highly profitable first-world companies will sometimes be able to pay third-world labourers meagre wages for arduous work without thereby exploiting them (Snyder 2010). Similarly, if DTC-GT companies purchase genetic data from individuals in third-world countries (for example, in order to reduce costs or include ancestry groups that are currently under-studied), a competitive market price might, intuitively, seem exploitatively low.

One could adopt a more demanding theory of exploitation. Robert Goodin (1987, 183), for example, argues that exploitation consists in “playing for advantage in situations where it is inappropriate to do so”—which might include pressing one’s advantage over victims of injustice. Similarly, Ruth Sample (2003) argues that exploitation consists in advantage-taking that is degrading towards the exploitee. Like Goodin, Sample argues that it is possible to exploit others by taking advantage of background injustice to offer a lower price than one would pay under fairer background conditions. If such theories are correct, DTC-GT companies might sometimes need to pay more than a competitive market price to avoid exploiting sellers.

Regardless of which theory of exploitation one adopts, DNA markets are not *inherently* exploitative; they are exploitative only if the benefits to sellers are unfairly low. This raises difficult philosophical questions about what would constitute a fair price, particularly when wealthy DTC-GT companies buy from sellers in the third world. While these questions deserve careful consideration, it is worth noting that they are not unique to DNA markets; they apply to all transactions between wealthy companies and people living in poverty. The general question here is whether it is exploitative for companies to take advantage of international inequality or injustice. Whatever conclusion one reaches, this conclusion will apply to many kinds of international markets; at least from the standpoint of exploitation, DNA markets specifically do not seem to raise any unique issues in this respect.

## Commodification

One final set of concerns centre on wrongful commodification. On one view—which seems to lurk behind some existing criticisms of DNA markets (see, e.g., Regalado 2016)—DNA markets commodify something that ought not to be treated as a commodity. To treat some good as a commodity is to treat it as though it is fungible*—*i.e., as though its value can be completely captured by its market price. As Margaret Radin puts it:

A fungible object can pass in and out of the person’s possession without effect on the person as long as its market equivalent is given in exchange; trading commodified objects is just like trading money. (Radin 1996, 87)

Commodification is not always morally problematic. Some things—like bricks and watermelons and pencils—are properly regarded as commodities. The question, then, is whether the commodification of *DNA* raises any unique moral issues.^5^

In a discussion of gene patenting, Stephen Wilkinson (2003) has outlined two possible reasons for thinking DNA should not be commodified: first, because DNA *itself* has cultural and symbolic value which cannot be reduced to economic value, and second, because selling DNA would treat persons as commodities. We consider both reasons in turn below.

## Commodification of DNA

It might be thought that DNA *itself* is not the kind of thing that ought to be commodified—that DNA has intrinsic value and should therefore not be treated as though it merely has a market price. There are two main forms this argument might take. The first holds that DNA has intrinsic value because it is so closely connected to human personhood. The second—which we return to below—appeals to DNA’s symbolic significance.

Does DNA have intrinsic value by dint of its association with human persons? Wilkinson (2003, 209–212) considers a number of reasons one might think that the intrinsic value of persons comes to rest in their DNA, but finds all of them problematic. It might be thought that if a person has value, then all parts of them have value (including their DNA), but this seems implausible because it is entirely possible for a part of an object to not share the properties of the whole object; if a book is heavy, it does not follow that a single page of that book is also heavy. A second interpretation come from identity; here, the idea is that having *my* genome is precisely what it means to be *me*. But this also seems false. Not only does this view neglect the crucial role of the environment, it also implies that two persons with the same genome—such as identical twins—share the same identity. This, too, seems implausible. Although human persons have intrinsic value, their DNA does not.

A second version of this argument might hold that DNA has special value by dint of its cultural and/or symbolic importance. DNA might be thought to represent what it means to be human and unites us as part of our common heritage. Given its symbolic significance, it might seem inappropriate to treat DNA as a mere commodity.

The first thing to note about this argument is that it depends on empirical facts about how people happen to regard DNA. Clearly, not all people tend to regard DNA as the kind of thing that ought not be bought or sold. To the contrary, it is sometimes argued that DNA donors *ought* to be rewarded for their contribution (DeFrancesco and Klevecz 2019). Even if people do currently attach symbolic significance to DNA, these views are not necessarily immutable. As Nussbaum (1998) has pointed out, a mere two hundred years ago it was thought inappropriate for artists, singers, actors, and dancers to receive payment for their work. Views about symbolic significance can transform over time. Indeed, if current views about symbolic significance prevent us from realizing important goals (like medical advancement), we might have moral reasons to try to bring about this transformation (Brennan and Jaworski 2015; Koplin 2018).

Assuming that DNA does have important symbolic value, we would further need to consider whether symbolic value provides legitimate grounds to abstain from (or legislate against) practices that are inconsistent with this symbolic value. To violate symbolic value might be *offensive*, but the mere fact that some practice is offensive is not usually thought to provide reason to block that practice. As Wilkinson (2003, 213) puts the point:

[I]f the mere fact that most people are offended by the existence of a practice is supposed to justify a ban on that practice, then given (for example) a widespread taboo regarding homosexuality, such an argument could be (mis)used in an attempt to justify banning same-sex sex. Indeed… arguments from actual offence could be used to justify bans on absolutely anything, as long as enough people were in fact offended.

In short, symbolic value provides a shaky basis for opposing DNA markets, as does the idea that DNA itself has intrinsic value. Pending some further argument to the contrary, the commodification of DNA per se does not seem to be morally problematic.

## Commodification of Persons

We found it difficult to see commodification of *DNA* as a moral problem. The commodification of *persons* is a different matter. It is widely thought that treating persons as commodities is offensive to their intrinsic value. As described above, pure commodities are mere things; their value is wholly captured by their market price. Persons have value beyond their market price; accordingly, they should not be treated as mere commodities. If DNA markets commodify *persons,* then this would provide a powerful argument against them.

Buying DNA would commodify sellers if the seller’s intrinsic value rests in their DNA. But as we explored in the preceding section, it is difficult to see why DNA should be afforded the same value as persons themselves. Since persons are not identical with their DNA, it is doubtful that DNA markets involve the literal commodification of persons.

Alternatively, it might be thought that paying for DNA would *tend to promote* the view that (some) persons lack intrinsic value. The argument here is that if we come to understand parts of humans as reducible to a market price, it may tend to promote the view that we can treat other persons—and not merely their genetic material—as a mere means to our own ends. Establishing a market in DNA samples risks eroding the view that all persons have inherent human dignity.

This argument echoes an existing criticism of DNA patents. This criticism runs as follows: since human DNA patenting uses market rhetoric to describe something intimately connected with human identity, it may promote the view that humans themselves (and not merely their DNA) are properly understood in terms of their market worth, not their intrinsic moral value (Resnik 2001). A similar argument has been raised against markets in human organs. Here, the idea is that valuing human body parts in terms of their market price might tend to promote the view that we can treat other persons—and not merely their “spare” kidneys—as a mere means to our own ends (Alpinar-Şencan, Baumann, and Biller-Andorno 2017; Kerstein 2009).

We can now see the shape of a new objection to DNA markets. Our DNA are closely linked to our identity. Indeed, for better or worse, the human genome is often described as *the* most central facet of who we are (Lewontin 1992). Accordingly, to treat our genetic information as the kind of thing that can be bought and sold might encourage some people to see *persons—*and not merely their DNA—as fungible commodities. By paying people for their genetic data, we risk eroding the view that all persons have intrinsic moral worth. Or so the argument might run.

One possible response to this argument points out that DNA is *already* commodified. After all, DTC-GT companies are already selling access to people’s genetic and personal information. To pay people for this data would not commodify something that currently exists outside the scope of the market; instead, it would serve to make the existing degree of commodification more transparent. From the perspective of informed consent, this transparency would arguably be a good thing.

We do not think this response succeeds. Offering payments for DNA might not introduce commodification where there was none before, but it would presumably increase the *degree* to which DNA (and related personal data) are commodified. This model would encourage donors—and not just DTC-GT companies—to regard their DNA as a commodity. If we are worried that commodifying DNA would erode respect for human persons, then presumably we should try not to increase the number of people who regard DNA in this way.

An alternative response might not reject the argument outright but instead question its force. After all, whether (or how fully) we commodify DNA is only one of myriad practices that might affect how persons view each other (Resnik 2001). The force of this kind of commodification concern turns on two questions—one empirical and one philosophical. First, to what extent would commodifying DNA erode human dignity? And second, how much weight should we attach to preserving dignity relative to other valuable goals, such as promoting human health? While we do not offer a full analysis of the issue here, we do not think it is obvious that commodifying DNA *would* greatly erode respect for persons, nor that the promotion or preservation of respect for persons should trump the other values at play. Concerns about eroding dignity (in the sense of eroding the view that all persons have intrinsic moral worth) do not provide a decisive reason against paying sellers, particularly if these payments would promote morally important goals.

## Conclusion

Existing ethical analyses of DNA markets have not explored the ethics of paying individuals for DNA samples. We have attempted to map the ethical terrain. Introducing payments could achieve some valuable goals, such as increasing the size of, and diversity within, genomic databases. They might also help make the true business model of DTC-GT companies clearer to consumers. With these advantages come additional concerns: that paying for samples may crowd out more altruistic incentives, introduce the possibility of undue inducement or exploitation, and/or commodify something that ought not be treated as a commodity.

While we do not find any of these concerns especially persuasive, there is room for further empirical and ethical analyses of each of these issues. For example, it would be useful to better understand how offering payments would affect altruistic donations and/or the quality of personal data, what constitutes a non-exploitative price for DNA, and how much force commodification concerns have in this context. However, as it stands, we think there are no decisive arguments against paying for genetic data. While there are moral limits to the market, our DNA probably falls outside them.

## Footnotes

1 A recent paper by Eman Ahmed and Mahsa Shabani (2019) is an important exception. However, where Ahmed and Shabani canvas ethical concerns regarding how DNA data is shared and how individuals’ control over the data is managed, we focus specifically on those ethical issues associated with *paying* individuals for access to their DNA data.

2 Indeed, this practice has already prompted some media backlash. For example, writing in *The Guardian,* Laura Spinney (2020) has written that “DNA testing companies are starting to profit from selling our data on to big pharma,” while a 2018 piece on the risks of DTC-GT published on CNBC asks, provocatively, “Who may profit on your DNA? The answer: Not you” (Rosenbaum 2018).

3 We leave open the question of whether payment would, in fact, provide an especially useful means for attracting donations of genetic data outside of the DTC-GT context. This is an empirical question that we think warrants further study.

4 For Wertheimer, such a price represents the threshold at which neither party takes *special* unfair advantage of the other party’s decision-making capacity or situation. A “fair price,” here, is defined within the context set by the prevailing market conditions (Wertheimer 1996, 232).

5 Similar concerns might be raised about the commodification of personal data provided by DNA donors*.* While these concerns are relevant to DNA markets, they are not exclusive to them; these concerns also apply (arguably more forcefully) to the use of online data for behavioural advertising (see, e.g., Roessler 2015). We focus here on the commodification of DNA (rather than personal data more generally) because we are especially interested in issues that are unique to DNA markets.
